# Supplementary material for: EnzML: multi-label prediction of enzyme classes using InterPro signatures
Source: BMC Bioinformatics. 2012 Apr 25;13:61. doi: 10.1186/1471-2105-13-61 (PMC3483700; doi:10.1186/1471-2105-13-61)
Supplement: Addtional file 5 — The Java code to format the data files, evaluate and predict. The file enzml_java_code.tar.gz contains the Java code used to format database data to ARFF and XML formats, to execute cross and train-test (jackknife) evaluations and to record evaluation results to database. More information is included in the readme.txt file and the Javadoc files. The code can be used with a MySQL database. To use a different database software, other JDBC drivers might be required. [file 1471-2105-13-61-S5.gz › java_code/enzml2011/doc/uk/ac/ed/inf/csb/taweka/ArffGenerator.html]

ArffGenerator


---


|  |  |  |
| --- | --- | --- |
| **Overview** \| **Package** \| **Class** \| **Use** \| **Tree** \| **Deprecated** \| **Index** \| **Help** | |  |
| PREV CLASS   **NEXT CLASS** | **FRAMES**    **NO FRAMES**     **All Classes** |  |
| SUMMARY: NESTED \| FIELD \| CONSTR \| METHOD | DETAIL: FIELD \| CONSTR \| METHOD |  |


---


## uk.ac.ed.inf.csb.taweka Class ArffGenerator

```
java.lang.Object
  uk.ac.ed.inf.csb.taweka.ArffGenerator
```

**Direct Known Subclasses:**: MulanArffGenerator

---

``` public class ArffGenerator extends java.lang.Object ```

General information about TaWeka (author, documentation web page, version
etc)

**Version:**
:   22 Apr 2010

**Author:**
:   Luna De Ferrari luna.deferrari-at-ed.ac.uk

---

| **Field Summary** | |
| --- | --- |
| `static java.lang.String` | `ARFF_TABLE`             The name of the table to save the arff file details to |
| `static java.lang.String` | `AUTHOR` |
| `static java.lang.String` | `TAWEKA_NAME` |
| `static java.lang.String` | `VERSION` |
| `static java.lang.String` | `WEB_PAGE` |


**Constructor Summary**

`ArffGenerator(java.lang.String dataPropsFileName,
java.lang.String resultPropsFileName)`


| **Method Summary** | |
| --- | --- |
| `void` | `generateArffFileFromDb()`             The core of TaWeka: save properties to experiment table, load data from data database and save arff to file |
| `java.lang.String` | `getArffPath()` |
| `ArffProperties` | `getDataArffProperties()` |
| `uk.ac.ed.inf.csb.utils.database.DbManager` | `getDataDbManager()` |
| `uk.ac.ed.inf.csb.utils.database.DbConn` | `getDbConn()` |
| `DbDataLoader` | `getDbLoader()` |
| `uk.ac.ed.inf.csb.utils.database.DbReader` | `getDbReader()` |
| `uk.ac.ed.inf.csb.utils.database.DbManager` | `getResultsDbManager()` |
| `boolean` | `isInitialisationIsCorrect()` |
| `void` | `setInitialisationIsCorrect(boolean initialisationIsCorrect)` |

**Methods inherited from class java.lang.Object**

`equals, getClass, hashCode, notify, notifyAll, toString, wait, wait, wait`

**Field Detail**

### ARFF\_TABLE

```
public static final java.lang.String ARFF_TABLE
```

:   The name of the table to save the arff file details to

    **See Also:**: Constant Field Values

---


### AUTHOR

```
public static final java.lang.String AUTHOR
```

**See Also:**: Constant Field Values

---


### TAWEKA\_NAME

```
public static final java.lang.String TAWEKA_NAME
```

**See Also:**: Constant Field Values

---


### VERSION

```
public static final java.lang.String VERSION
```

**See Also:**: Constant Field Values

---


### WEB\_PAGE

```
public static final java.lang.String WEB_PAGE
```

**See Also:**: Constant Field Values


**Constructor Detail**

### ArffGenerator

```
public ArffGenerator(java.lang.String dataPropsFileName,
                     java.lang.String resultPropsFileName)
```


**Method Detail**

### generateArffFileFromDb

```
public void generateArffFileFromDb()
```

:   The core of TaWeka: save properties to experiment table, load data from
    data database and save arff to file

---


### getArffPath

```
public java.lang.String getArffPath()
```

---


### getDataArffProperties

```
public ArffProperties getDataArffProperties()
```

---


### getDataDbManager

```
public uk.ac.ed.inf.csb.utils.database.DbManager getDataDbManager()
```

---


### getDbConn

```
public uk.ac.ed.inf.csb.utils.database.DbConn getDbConn()
```

---


### getDbLoader

```
public DbDataLoader getDbLoader()
```

---


### getDbReader

```
public uk.ac.ed.inf.csb.utils.database.DbReader getDbReader()
```

---


### getResultsDbManager

```
public uk.ac.ed.inf.csb.utils.database.DbManager getResultsDbManager()
```

---


### isInitialisationIsCorrect

```
public boolean isInitialisationIsCorrect()
```

---


### setInitialisationIsCorrect

```
public void setInitialisationIsCorrect(boolean initialisationIsCorrect)
```


---


|  |  |  |
| --- | --- | --- |
| **Overview** \| **Package** \| **Class** \| **Use** \| **Tree** \| **Deprecated** \| **Index** \| **Help** | |  |
| PREV CLASS   **NEXT CLASS** | **FRAMES**    **NO FRAMES**     **All Classes** |  |
| SUMMARY: NESTED \| FIELD \| CONSTR \| METHOD | DETAIL: FIELD \| CONSTR \| METHOD |  |


---
